# O-GlcNAcylation at S659 enhances SARS-CoV-2 spike protein stability and pseudoparticle packaging efficiency

**DOI:** 10.1128/spectrum.00527-25

**Published:** 2025-07-28

**Authors:** Ting Xu, Jie Li, Xiaoxuan Lu, Shuai Song, Shengnan Wang, Jing Li, Leiliang Zhang

**Affiliations:** 1 Department of Clinical Laboratory Medicine, The First Affiliated Hospital of Shandong First Medical University & Shandong Provincial Qianfoshan Hospital 66310, Jinan, Shandong, China; 2 Department of Pathogen Biology, School of Clinical and Basic Medical Sciences, Shandong First Medical University & Shandong Academy of Medical Sciences 518873, Jinan, Shandong, China; 3 Beijing Key Laboratory of DNA Damage Response and College of Life Sciences, Capital Normal University 12379, Beijing, China; Universiteit Utrecht, Utrecht, the Netherlands

**Keywords:** SARS-CoV-2, spike protein, O-GlcNAcylation, ubiquitination, virus entry

## Abstract

The spike (S) protein of severe acute respiratory syndrome coronavirus 2 (SARS-CoV-2) plays a crucial role in viral entry and pathogenesis, making it an important target for therapeutic strategies. In this study, we explore the interaction between the S protein and O-GlcNAc transferase (OGT), revealing a physical association between these proteins using immunoprecipitation and glutathione-S-transferase (GST)-pulldown assays. Our results demonstrate that Serine 659 (S659) is the primary site of O-GlcNAcylation on the S protein, as confirmed by ion mobility mass spectrometry and mutagenesis studies. Notably, the S659A mutation significantly reduces O-GlcNAcylation of the S protein, leading to increased ubiquitination and subsequent degradation of the S protein. Cycloheximide pulse-chase assays further corroborate that the wild-type S protein exhibits greater stability compared with the S659A mutant of S. Despite these stability changes, the S659A mutation does not impair the binding affinity of the S protein to its receptor ACE2. However, we find that the S659A mutation significantly decreases the packaging efficiency of SARS-CoV-2 pseudoparticles. Collectively, our findings highlight the critical role of O-GlcNAcylation at S659 in regulating S protein stability and viral particle assembly, underscoring its potential as a target for therapeutic intervention against SARS-CoV-2.

**IMPORTANCE:**

This study highlights the critical role of the spike (S) protein of severe acute respiratory syndrome coronavirus 2 (SARS-CoV-2) in the viral infection process and its potential as a target for therapies and vaccines. By identifying Serine 659 (S659) as a key site for O-GlcNAcylation, the research reveals how modifications at this residue can influence the protein’s interactions with host factors, thereby affecting viral replication and pathogenicity. Furthermore, the S659A mutation was shown to lead to a significant increase in ubiquitination and degradation of the S protein, indicating that O-GlcNAcylation is crucial for modulating the protein’s stability and, consequently, its efficiency in facilitating viral entry. Understanding these mechanisms is vital for the development of effective interventions against coronavirus disease 2019 (COVID-19). Overall, this research enhances our understanding of how post-translational modifications impact viral behavior, opening avenues for innovative strategies to combat SARS-CoV-2 and future viral threats.

## INTRODUCTION

The emergence of severe acute respiratory syndrome coronavirus 2 (SARS-CoV-2) has led to a global pandemic, significantly impacting public health and economies worldwide (1–4). Central to the pathogenesis of this virus is its spike (S) protein, which is essential for viral entry into host cells (5, 6). This protein facilitates the binding of the virus to angiotensin-converting enzyme 2 (ACE2) on the surface of human cells, marking a critical step in the initiation of infection (7–10). Understanding the molecular mechanisms that regulate the structure and function of the S protein is crucial for developing therapeutics and vaccines aimed at mitigating the spread of this virus.

Recent studies have highlighted the intricate glycosylation patterns of the S protein, which undergoes extensive post-translational modifications, including O-glycosylation and N-glycosylation (11–13). These modifications extend beyond mere structural changes; they play significant roles in influencing the biological functions of the S protein. For instance, polypeptide N-acetylgalactosaminyltransferase 3 (GalNAc-T3) and GalNAc-T7 mediate the clustered O-GalNAc type glycosylation events in the S protein (11). This O-GalNAc type glycosylation is critical for the subsequent furin processing of the S protein, a critical step for virion assembly (11). In another study, S with N-glycosylation defect has been reported to reduce SARS-CoV-2 infection *in vitro* (14). Modifying the N-glycosylation of newly synthesized S protein impairs the maturation and release of infectious virions, resulting in the secretion of fewer virions with missing or aberrant spike N-glycans crucial for cellular invasion, thus rendering a portion noninfectious. The dynamic interplay between these glycosylation modifications highlights their importance in the functionality and pathogenicity of the S protein.

Among the various types of glycosylation, O-GlcNAcylation is a unique and critical modification characterized by the attachment of O-linked β-N-acetylglucosamine (O-GlcNAc) to serine or threonine residues on proteins (15). This modification is tightly regulated by two key enzymes: O-GlcNAc transferase (OGT), which catalyzes the addition of the O-GlcNAc moiety, and O-GlcNAc hydrolase (OGA), which removes it (16). The role of O-GlcNAcylation in the context of viral proteins and their interactions with host cellular mechanisms remains an active area of investigation. Notably, the implications of O-GlcNAc modifications on the S protein of SARS-CoV-2 are not yet fully understood, thus leaving a significant gap in our knowledge of the virus’s biology.

In this study, we investigate the Serine 659 (S659) site of the SARS-CoV-2 S protein and its potential O-GlcNAc modification. Using biochemical and biophysical methods, we examine how this modification affects the structural integrity and functional properties of the S protein. Insights gained from analyzing this site may uncover new therapeutic targets and enhance our understanding of SARS-CoV-2 biology, which could inform the development of effective interventions against coronavirus disease 2019 (COVID-19).

## RESULTS

### Interaction of S protein with OGT

**Fig 1:**
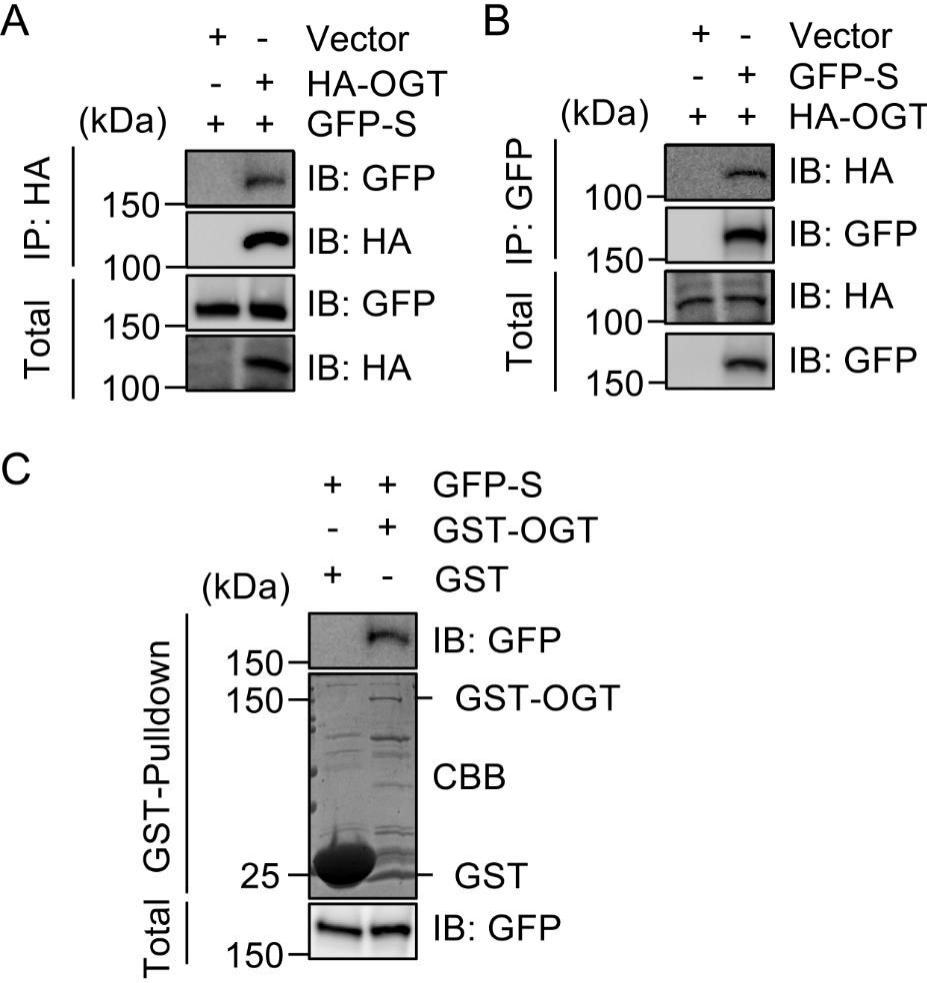
S protein from SARS-CoV-2 associates with OGT. (**A**) HEK293T cells were transfected with the plasmids expressing GFP-S and HA-OGT or empty vector. The cell lysates were subject to IP and immunoblotting with the antibodies indicated. (**B**) HEK293T cells were transfected with the plasmids expressing HA-OGT and GFP-S or empty vector. The cell lysates were subject to IP and immunoblotting with the antibodies indicated. (**C**) HEK293T cells were transfected with GFP-S plasmids, and then the lysates were incubated with recombinant GST-OGT proteins purified from *E. coli*, and GST-pulldown assays were carried out.

To investigate the interaction between the S protein from the Wuhan-Hu-1 strain of SARS-CoV-2 and OGT, we performed immunoprecipitation (IP) experiments. Initially, HEK293T cells were transfected with plasmids encoding hemagglutinin (HA)-tagged OGT and green fluorescent protein (GFP)-tagged S protein. The cell lysates were subsequently subjected to reciprocal IP using specific antibodies against HA and GFP. Immunoblot analysis revealed a significant association between the S protein and OGT (Fig. 1A and B), thereby supporting the hypothesis that these proteins engage in a physical association within the cellular context.

To further validate this interaction, we conducted GST-pulldown assays using recombinant GST-OGT proteins purified from *Escherichia coli*. HEK293T cells were transfected with GFP-S plasmids, and the resulting cell lysates were incubated with either GST-OGT or GST control proteins. The presence of GFP-tagged S protein was effectively detected in samples pulled down by GST-OGT, while no such interaction was observed with the GST control, confirming the specificity of the interaction (Fig. 1C).

These findings strongly indicate that the S protein of SARS-CoV-2 interacts directly with OGT, suggesting a potential role of OGT in the post-translational modification and functional regulation of the viral S protein. This interaction may provide valuable insights into the molecular mechanisms underlying SARS-CoV-2 pathogenesis and could inform the development of targeted therapeutic strategies against the viral lifecycle.

### O-GlcNAcylation modification of SARS-CoV-2 S protein at S659

**Fig 2:**
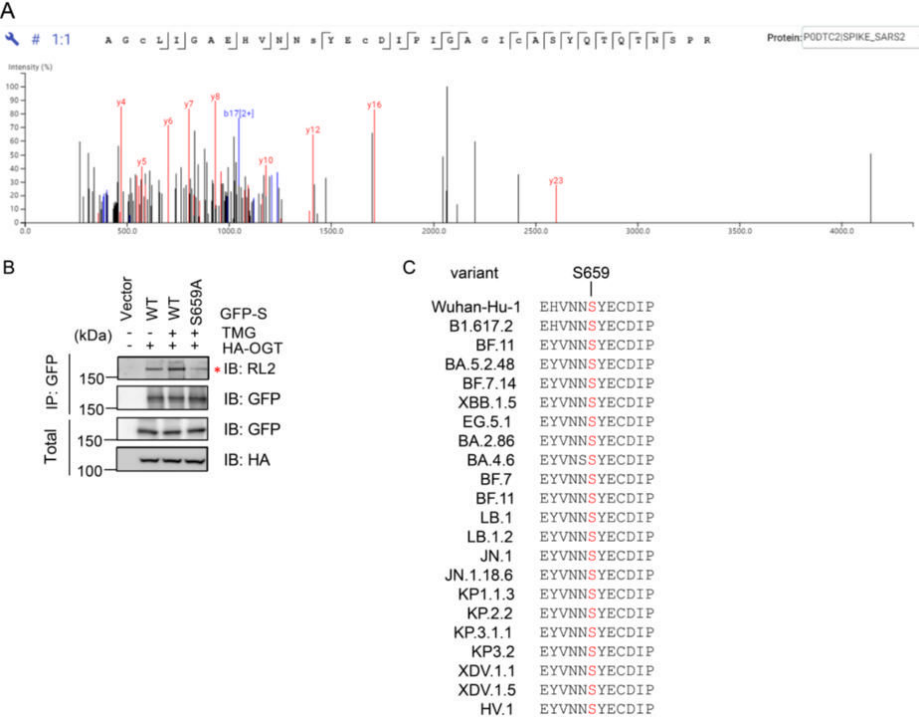
S protein from SARS-CoV-2 is O-GlcNAcylated at S659. (**A**) Ion mobility mass spectrometry identifies an O-GlcNAcylated peptide on the S protein. Ion mobility mass spectrometry was performed by Applied Protein Technology, Shanghai. The O-GlcNAc site of S was identified by PEAKS studio. (**B**) HEK293T cells were transfected with plasmids expressing GFP-S-WT, GFP-S-S659A, or empty vector together with HA-OGT, treated or untreated with the OGA inhibitor Thiamet-G (TMG) to enrich O-GlcNAcylation. Then, the cell lysates were immunoprecipitated with anti-GFP antibodies and immunoblotted with anti-O-GlcNAc RL2 antibodies. * indicated the position of the target protein. (**C**) Ser-659 is evolutionarily conserved among various variants of SARS-CoV-2.

To investigate the specific site(s) of O-GlcNAcylation on the SARS-CoV-2 S protein, we utilized ion mobility mass spectrometry, which is a powerful technique for analyzing post-translational modifications. In our mass spectrometry analysis, we detected a peptide containing S659 (AGCLIGAEHVNNS*YECDIPIGAGICASYQTQTNSPR, with * denoting the modification site) that exhibited a significant mass shift of +203.07 Da, which is consistent with the theoretical value of O-GlcNAc modification (Fig. 2A). The fragment ion spectrum (specifically, the b17 ion) of this peptide clearly indicates that the modification site is located at S659 (Fig. 2A). This finding was further supported by mutagenesis studies, in which HEK293T cells were transfected with plasmids expressing either GFP-tagged wild-type S (GFP-S-WT) or a mutated version, GFP-S-S659A, along with HA-tagged OGT. Cells were treated with the O-GlcNAc hydrolase inhibitor Thiamet-G (TMG) to enhance the detection of O-GlcNAcylation. Subsequent IP using anti-GFP antibodies followed by Western blot analysis with anti-O-GlcNAc RL2 antibodies demonstrated a significant enrichment of O-GlcNAcylated S protein in the WT construct, whereas the S659A mutation led to a marked reduction in O-GlcNAc signal, confirming the importance of this residue for O-GlcNAcylation (Fig. 2B).

Moreover, we assessed the evolutionary conservation of S659 across known variants of SARS-CoV-2. Sequence alignment analysis revealed that the serine residue at this position is consistently maintained among emerging variants, underscoring the functional significance and evolutionary conservation of O-GlcNAcylation at this site (Fig. 2C).

These findings collectively suggest that S659 serves as a critical site for O-GlcNAcylation on the SARS-CoV-2 S protein. This post-translational modification may play an essential role in the biology of the virus, influencing aspects, such as viral replication or host interaction, thereby highlighting S659 as a potential target for future therapeutic strategies against SARS-CoV-2.

### Impact of O-GlcNAcylation on S protein stability

**Fig 3:**
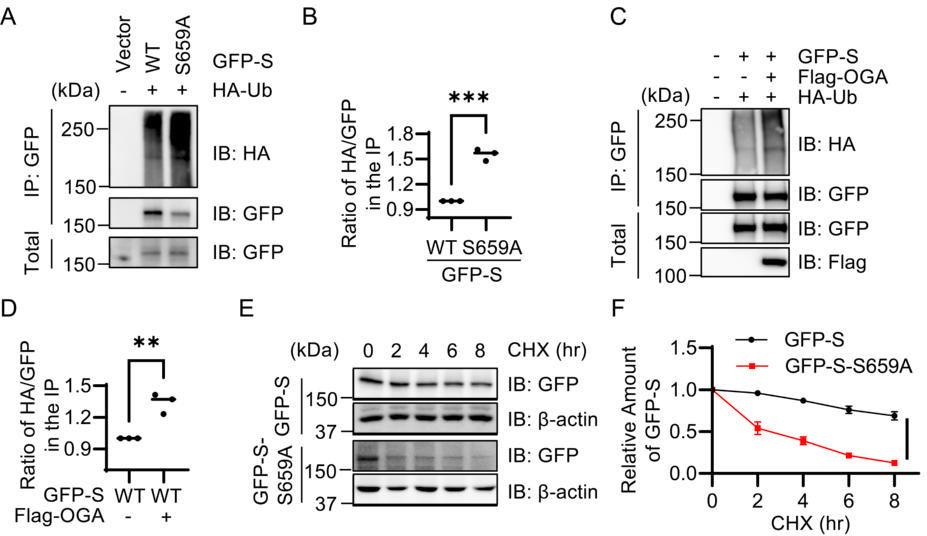
O-GlcNAcylation of S at S659 regulates S protein stability. (**A**) HEK293T cells were transfected with the plasmids expressing GFP-S-WT, -S659A, or empty vector, and the lysates were immunoprecipitated with anti-GFP antibody and immunoblotted with indicated antibodies. (**B**) Quantitation of (**A**). (**C**) HEK293T cells were transfected with the plasmids expressing GFP-S-WT and HA-Ub together with Flag-OGA or mock. The lysates were immunoprecipitated with anti-GFP antibody and immunoblotted with indicated antibodies. (**D**) Quantitation of (**C**). (**E-F**) Cycloheximide (CHX) pulse-chase assays. HEK293T cells were transfected with GFP-S-WT or -S659A plasmids, and then treated with CHX for different durations (**E**). The quantitation is in panel **F**. **, *P* < 0.01; ***, *P* < 0.001.

Next, we assessed the role of O-GlcNAcylation in influencing the stability of the S protein, given its recognized interplay with ubiquitination processes (17). Our co-transfection experiments revealed that the S protein variant lacking O-GlcNAc modification, specifically the S659A mutant, exhibited significantly increased levels of ubiquitination (Fig. 3A and B). This observation suggests that the absence of O-GlcNAcylation may predispose the S protein to more extensive ubiquitin-mediated degradation pathways.

To reinforce our findings, we next examined the effects of overexpressing Flag-tagged O-GlcNAc transferase (Flag-OGA). Our results indicated that the overexpression of Flag-OGA resulted in further elevations in the ubiquitination levels of the S protein (Fig. 3C and D). These data collectively imply that O-GlcNAcylation is indeed a crucial post-translational modification that contributes to the stabilization of the S protein by mitigating ubiquitination-dependent degradation processes.

To quantitatively assess the stability of the S protein variants, we utilized cycloheximide (CHX), a potent translation inhibitor (18), and conducted CHX pulse-chase assays (19). HEK293T cells transfected with either GFP-S-WT or GFP-S-S659A plasmids were treated with CHX for varying time intervals to halt protein synthesis. Analysis of the results demonstrated that the wild-type S protein (S-WT) exhibited a significantly prolonged half-life compared with the S659A mutant (Fig. 3E and F). This notable difference in stability underscores the critical role that O-GlcNAcylation plays in maintaining the integrity and functionality of the S protein within the cellular environment.

### Effect of S-659A mutation on viral entry

**Fig 4:**
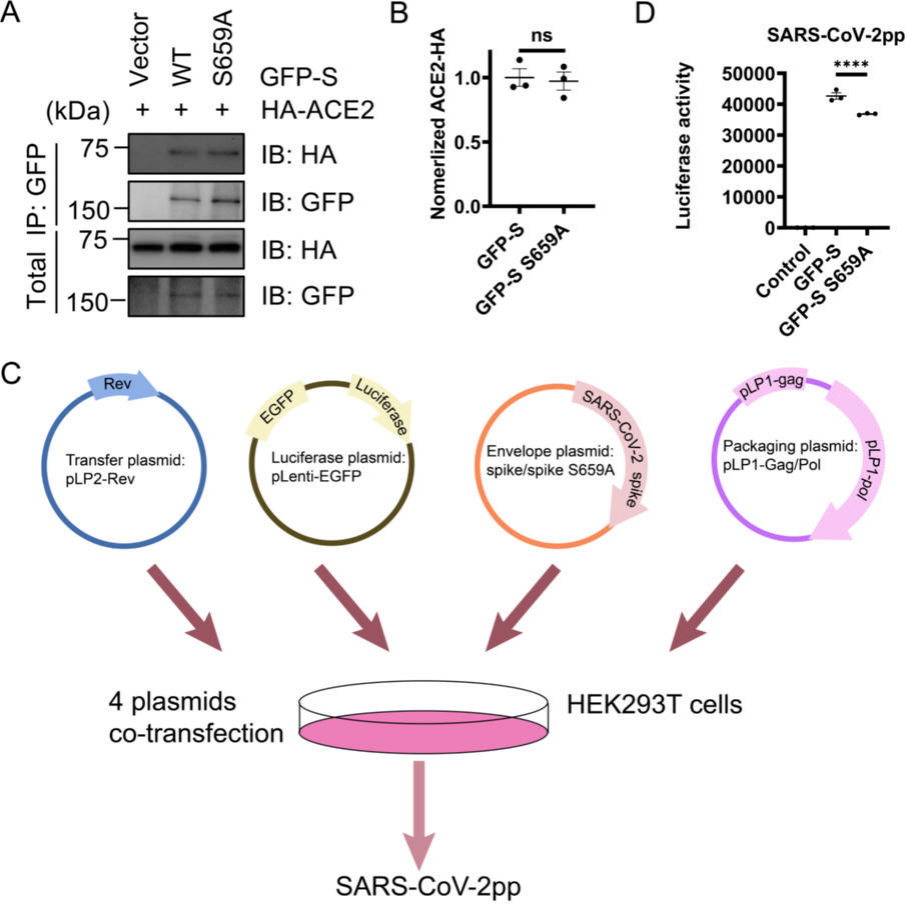
O-GlcNAcylation of S at S659 regulates pseudoviral packaging of SARS-CoV-2. (**A**) S-S659A does not affect the binding between S and Ace2. HEK293T cells were transfected with the plasmids expressing HA-ACE2 and GFP-S-WT, GFP-S-659A, or empty vector. The cell lysates were subject to IP. (**B**) Quantitation of (**A**). (**C**) Diagram of four-plasmid system for SARS-CoV-2pp production. The four-plasmid system for preparing pseudovirus SARS-CoV-2pp includes Luc (pLenti6) encoding luciferase, HIV Gag/pol (pLP1), HIV Rev (pLP2), and GFP-S. (**D**) S659A mutation in SARS-CoV-2 S reduced the SARS-CoV2pp package efficiency. The luciferase activity of lysates from Huh7.5.1 cells infected with SARS-CoV-2pp generated from HEK293T cells based on either S or the S-S659A was assayed. ns, no significance; ****, *P* < 0.0001.

To further elucidate the role of the S-659A mutation in the context of viral entry, we measured the affinity of the mutated S protein for its primary receptor, ACE2. Our analysis revealed that there were no significant differences in binding affinity when comparing the S-659A mutant to the S-WT (Fig. 4A and B). This result suggests that the alteration at position 659 does not impair the interaction between the S protein and ACE2, a critical step for viral entry.

In order to evaluate the impact of the S-659A mutation on the packaging efficiency of SARS-CoV-2, we employed a four-plasmid system to produce SARS-CoV-2 pseudoparticles (SARS-CoV2pp). This system involved the co-expression of luciferase (pLenti6), HIV Gag/pol (pLP1), HIV Rev (pLP2), and the S protein (Fig. 4C). Upon analyzing the luciferase activity in Huh7.5.1 cells that were infected with SARS-CoV2pp generated from HEK293T cells, we observed a marked decrease in luciferase signals for pseudoparticles containing the S-S659A mutant (Fig. 4D). This reduction in luciferase activity indicates a significant impairment in the packaging efficiency of SARS-CoV-2 when the S protein harbors the S-659A mutation. Collectively, these findings highlight the importance of the S659 site in viral particle assembly, even though the mutation does not affect the receptor-binding interaction.

## DISCUSSION

In this study, we investigated the role of O-GlcNAcylation at the S659 site of the SARS-CoV-2 S protein, exploring its interactions with OGT and its implications for protein stability and viral entry. Our findings confirm a significant association between the S protein from the Wuhan-Hu-1 strain and OGT, supported by reciprocal IP experiments and pull-down of GFP-tagged S proteins by GST-OGT (Fig. 1A, B and C). Notably, our identification of S659 as the key O-GlcNAcylation site using ion mobility mass spectrometry aligns with previous studies that have highlighted the importance of O-glycosylation in coronavirus biology (11, 20–22). Moreover, the evolutionary conservation of the serine residue at this position among emerging variants underscores the potential functional significance of this modification (Fig. 2C).

Our study further reveals that O-GlcNAcylation impacts the stability of the S protein. The observation that the O-GlcNAc-deficient S659A mutant exhibited increased ubiquitination suggests that O-GlcNAcylation may protect the S protein from proteasomal degradation (Fig. 3A and B). This finding is consistent with existing literature that has linked O-GlcNAc modification to enhanced protein stability through modulation of ubiquitination pathways (17). Additionally, the results from the CHX treatment indicate that wild-type S protein has a significantly extended half-life compared to the S659A mutant, emphasizing the role of O-GlcNAcylation in maintaining S protein stability (Fig. 3E and F).

While our findings provide valuable insights, we observed no significant differences in ACE2 binding affinity between the S-659A mutant and the WT S protein (Fig. 4A and B). The fact that the S659A mutation does not affect the binding of the S protein to ACE2 suggests that the structural integrity of the receptor-binding domain (RBD) remains intact, facilitating successful receptor engagement. However, our production of SARS-CoV-2pp using a four-plasmid system revealed a decrease in luciferase activity in Huh7.5.1 cells infected with the S-S659A mutant, indicating reduced packaging efficiency (Fig. 4D). This reduction in packaging efficiency implies that other factors may be involved, potentially including O-GlcNAc modification or conformational changes of the S protein that could affect its stability. One possible explanation is that, although the S protein can still bind to ACE2, the mutations might alter the protein’s stability or localization within the cell, adversely impacting its incorporation into new virions. For instance, alterations in glycosylation patterns, such as O-GlcNAcylation at S659, could influence the folding and trafficking of the S protein, ultimately affecting how efficiently it is packaged into mature virions. Moreover, it is essential to consider the role of other host factors in packaging efficiency. The interactions between the S protein and the cellular machinery involved in virion assembly and budding may be modified by the observed mutations, leading to changes in packaging efficiency despite an unchanged binding affinity.

**Fig 5:**
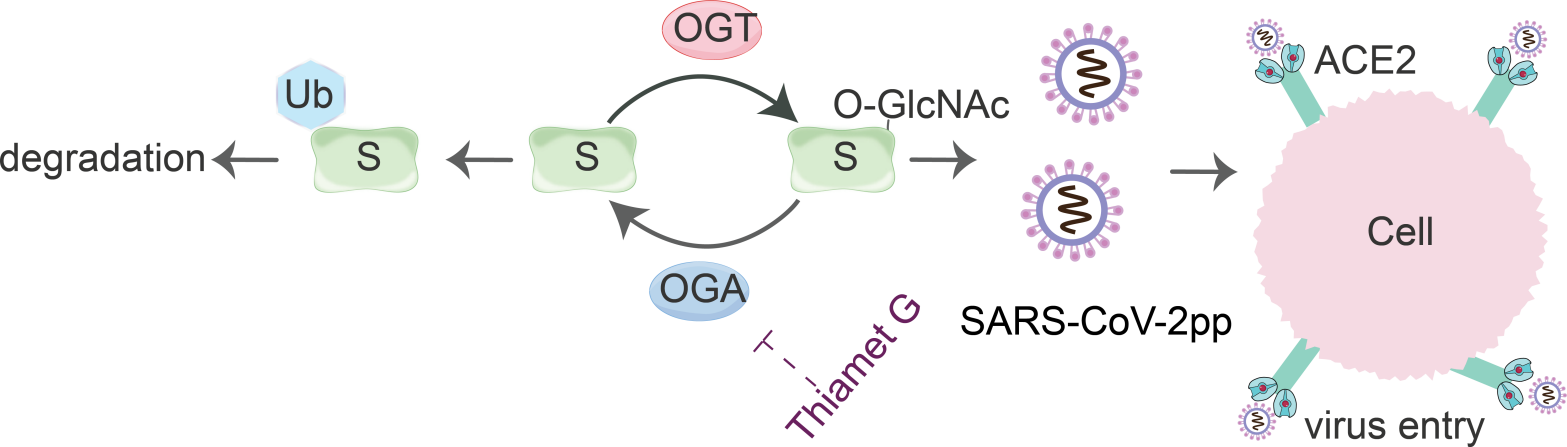
The model of O-GlcNAcylation of the SARS-CoV-2 S protein. The S protein is modified by O-GlcNAcylation, which is promoted by OGT and reduced by OGA. O-GlcNAcylation enhances the stability of the S protein, while non-GlcNAcylated S is targeted for ubiquitination and degradation. Thiamet G, an OGA inhibitor, elevates the levels of O-GlcNAcylation on the S protein. Proper glycosylation of the S protein is essential for the entry of SARS-CoV-2pp, which is mediated by the ACE2 receptor.

Our research highlights the critical role of O-GlcNAcylation at the S659 site in regulating the stability of the SARS-CoV-2 S protein (Fig. 5). We chose the Wuhan-Hu-1 strain of SARS-CoV-2 because it was the first strain identified during the COVID-19 pandemic. Notably, the S659 site remains evolutionarily conserved as SARS-CoV-2 undergoes mutations across various variants, suggesting that the regulatory mechanisms associated with this site are essential for the virus’s viability and potential pathogenicity. These findings deepen our understanding of viral biology by illuminating the intricate mechanisms that underlie the stability and function of the S protein, which is crucial for viral entry into host cells. Identifying S659 as a potential target for O-GlcNAcylation paves the way for developing novel antiviral strategies aimed at disrupting this modification, thereby affecting the functionality of the S protein. Furthermore, our research encourages further exploration of the interplay among various post-translational modifications that the S protein may experience, including phosphorylation, ubiquitination, and glycosylation. It is likely that these modifications, including O-GlcNAcylation at S659, engage in cross-talk, influencing each other’s effects on the stability and activity of the S protein. Such interactions could have significant implications for viral entry and pathogenesis, highlighting the need for future studies to take a more integrative approach that examines these multiple post-translational modifications in concert. Ultimately, a deeper understanding of these complex regulatory networks will enrich our collective knowledge of SARS-CoV-2 biology and could lead to innovative therapeutic interventions that address the intricate dynamics of viral infection and evolution.

Despite the strengths of our study, there are limitations that should be acknowledged. Our examination focused primarily on the S protein’s interaction with OGT and its stability; however, we did not investigate the potential effects of other post-translational modifications that may also impact S protein function. Furthermore, the use of SARS-CoV-2pp provides valuable insights into viral entry, yet studies using live virus models may further elucidate the biological relevance of our findings in a more physiologically relevant context.

In conclusion, the identification of highly conserved O-GlcNAcylation at residue S659 in the SARS-CoV-2 S protein highlights the critical role of glycosylation in viral pathogenesis. Our data suggest that this modification not only enhances the stability of the S but also improves the efficiency of pseudoviral particle packaging. Future therapeutic strategies targeting O-GlcNAcylation could offer novel approaches to combat SARS-CoV-2 and its variants, necessitating further exploration of targeted glycosylation inhibitors.

## MATERIALS AND METHODS

### Cell culture

HEK293 cell was purchased from COBIOER (CBP60232, Nanjing, China). Huh7.5.1 cell was a gift from Dr. Francis Chisari. The cell lines were validated using STR profiling and free from mycoplasma contamination for all experiments.

### Antibodies

The antibodies were anti-Flag (XHY021L, Beijing, China), rabbit anti-GFP (50430-2-AP, Proteintech, Chicago, USA), anti-HA (51064-2-AP, Proteintech, Chicago, USA), HRP-conjugated affinipure goat anti-rabbit IgG(H + L) (SA00001-2, Proteintech, Chicago, USA), anti-β-actin (YM3028, Immunoway, San Jose, CA, USA), anti-O-GlcNAc (RL2) (ab2739, Abcam, Waltham, MA, USA).

### Plasmids

The SARS-CoV-2 (2019-nCoV) Spike ORF cDNA expression plasmid with an N-GFP tag was purchased from Sino Biological (RG81059-ANG). The human ACE2 ORF cDNA clone expression plasmid with a C-HA tag was sourced from Sino Biological (HG10108-CY). The four-plasmid system for preparing SARS-CoV-2pp includes Luc (pLenti6) encoding luciferase, HIV Gag/pol (pLP1), and HIV Rev (pLP2) and was a gift from Dr. Ping Zhao from Navy Medical University. The plasmids expressing GFP-OGT, GFP-S-S659A, HA-Ub, HA-OGT, and Flag-OGA were cloned in our lab.

### IP and immunoblotting

The IP and immunoblotting assays were described before (23). The ECL detection system (Beyotiome, P0018FM-1, P0018FM-1, China) was used for immunoblotting. Bio-Rad ChemiDoc Touch was employed to detect signals, and the signals were quantitated by the Image J software. All Western blots were repeated for at least three times. Ionizing mobility mass spectrometry was performed by Applied Protein Technology, Shanghai.

### GST fusion protein preparation

The expression of the GST fusion protein was induced in *E. coli* Rosetta (DE3) by the addition of 0.5 mM isopropyl β-D-thiogalactoside (IPTG) and allowed to proceed at 37°C for 5 h. Following induction, bacterial cells were lysed using a lysis buffer containing 50 mM Tris (pH 6.8), 1 mM EDTA, and 100 mM NaCl. The GST fusion proteins were then purified with glutathione–Sepharose beads. For the pulldown assay, cell lysates were prepared in a GST pulldown buffer composed of 50 mM Hepes (pH 7.2), 150 mM NaCl, 90 mM KCl, 1 mM EDTA, and 0.5% NP-40, which were then incubated with the GST fusion proteins for 1 h at 4°C. After incubation, the glutathione beads were pelleted and washed three times with PBS buffer. The samples were subsequently analyzed by Western blotting.

### SARS-CoV-2pp packaging

For SARS-CoV-2pp production, on the day before transfection, HEK293T cells were seeded in plates at a density of 1.8 × 10^7^ cells per well. Co-transfection was performed by introducing plasmids expressing Luc (pLenti6), HIV Gag/Pol (pLP1), HIV Rev (pPL2), and GFP-S/GFP-S S659A (in a ratio of 8:4:3:3) into the HEK293T cells using PEI MAX-Transfection Grade Linear Polyethylenimine Hydrochloride (PEI, MW 40000, USA, 24765-1). After 48 h, the supernatants were collected into a 15 mL tube, centrifuged to remove precipitates, and the SARS-CoV-2pp was stored at −80°C for future use. For transduction, Huh7.5.1 cells were seeded in a 24-well plate 1 day prior. The medium was then replaced with DMEM without antibiotics and serum, followed by addition of the viral supernatant. After 2 days, the cells were washed, lysed, and the lysates were transferred to a 96-well plate. Subsequently, they were incubated with Steady-glo (Promega, Madison, USA), and luciferase activity was measured using a microplate reader (TECAN SPARK).

### Statistics

Statistical analyses were performed using GraphPad Prism software version 8.0 (GraphPad Software, Inc., La Jolla, CA, USA). To assess significant differences between the treated and control groups, a two-tailed Student’s *t*-test was employed. Results are reported as mean ± standard deviation. A *P*-value greater than 0.05 was deemed not statistically significant (ns). The significance levels are indicated as follows: *, *P* < 0.05; **, *P* < 0.01; ***, *P* < 0.001; ****, *P* < 0.0001.

## Data Availability

The data that support the findings of this study are available on request from the corresponding authors upon reasonable request.
